# Poloxamer-188 and d-α-Tocopheryl Polyethylene Glycol Succinate (TPGS-1000) Mixed Micelles Integrated Orodispersible Sublingual Films to Improve Oral Bioavailability of Ebastine; In Vitro and In Vivo Characterization

**DOI:** 10.3390/pharmaceutics13010054

**Published:** 2021-01-04

**Authors:** Nayyer Islam, Muhammad Irfan, Salah-Ud-Din Khan, Haroon Khalid Syed, Muhammad Shahid Iqbal, Ikram Ullah Khan, Amina Mahdy, Mohamed Raafat, Mohammad Akbar Hossain, Sana Inam, Rabia Munir, Memoona Ishtiaq

**Affiliations:** 1 Department of Pharmaceutics, Faculty of Pharmaceutical Sciences, Government College University Faisalabad, Faisalabad 38000, Pakistan; nayyerislam1@gmail.com (N.I.); syedharoonkhalid@gcuf.edu.pk (H.K.S.); ikramglt@gmail.com (I.U.K.), drsanainam@gmail.com (S.I.), rabiamunir_786@yahoo.com (R.M.), memoona62@yahoo.com (M.I.); 2 Department of Biochemistry, College of Medicine, Imam Mohammad Ibn Saud Islamic University (IMSIU), Riyadh 11432, Saudi Arabia; sdikhan@imamu.edu.sa; 3 Department of Clinical Pharmacy, College of Pharmacy, Prince Sattam bin Abdulaziz University, Alkharj 11942, Saudi Arabia; drmmsiqbal@gmail.com; 4 Pharmacology Department, International School of Medicine, Medipol University, Istanbul 34810, Turkey; aminamahdy@yahoo.com or; 5 Department of Pharmacology and Toxicology, College of Pharmacy, Umm Al Qura University, Makkah P.O. Box 715, Saudi Arabia; raafatabdalla@hotmail.com; 6 Department of Pharmacology and Toxicology, Faculty of Medicine, Umm Al Qura University, Makkah P.O. Box 715, Saudi Arabia; mahossain@uqu.edu.sa

**Keywords:** ebastine, poloxamer-188, d-α-tocopheryl polyethylene glycol succinate, micelles, bioavailability, toxicity

## Abstract

Orodispersible sublingual films (OSFs) composed of hydrophilic polymers were loaded with poloxamer-188 and d-α-tocopheryl polyethylene glycol succinate (TPGS-1000) mixed micelles to improve the oral bioavailability of a poorly soluble drug, ebastine (EBT). Mixed micelles formed by thin-film hydration method were incorporated into orodispersible sublingual film, consisting of HPMC and glycerol, using solvent casting technique. The mixed micelles and films were thoroughly evaluated for physicochemical characterization (size, polydispersity index, zeta potential, entrapment efficiency, thickness, weight, surface pH studies, disintegration time, swelling indices, mechanical properties, FTIR, PXRD, DSC, SEM, AFM, in vitro drug release, in vivo bioavailability, and toxicological studies). The results showed that the average particle size of mixed micelles was 73 nm. The mean zeta potential and PDI of the optimal mixed micelles formulation were −26 mV and 0.16, respectively. Furthermore, the maximum entrapment efficiency 82% was attained. The film’s disintegration time was in the range of 28 to 102 s in aqueous media. The integrity of micelles was not affected upon incorporation in films. Importantly, the micelles-loaded films revealed rapid absorption, high permeability, and increased bioavailability of EBT as compared to the pure drug. The existence of ebastine loaded mixed micelles in the films enhanced the bioavailability about 2.18 folds as compared to pure drug. Further, the results evidently established in-vitro and in-vivo performance of bioavailability enhancement, biocompatibility, and good safety profile of micelles-loaded orodispersible EBT films. Finally, it was concluded that film loaded with poloxamer-188/TPGS-1000 mixed micelles could be an effective carrier system for enhancing the bioavailability of ebastine.

## 1. Introduction

According to the literature, allergy bothers 10 to 30% of the worldwide population. Among available treatments for allergic diseases, ebastine (EBT) is one of the potent antagonist of H_1_ receptor that is used for treatment of a wide range of allergies [1]. EBT does not produce sedation as well as toxicity compared to other therapeutic treatments for allergies thus EBT is considered suitable candidate for antihistaminic commitments. Nevertheless, EBT provides low bioavailability owing to its poor solubility. EBT is practically insoluble in water [2]. Another reason for low oral bioavailability is extensive metabolism in the gastrointestinal tract and liver. The interactions of EBT in intestine along with its physicochemical properties are considered leading causes of its poor bioavailability [3]. The reported oral bioavailability of EBT in literature is around 40% [4].

Numerous approaches have been applied to improve the bioavailability of EBT through oral route. Although the oral route is most convenient for drug administration, EBT bioavailability was not improved convincingly with conventional drug delivery systems. Some of existing technologies and formulations used for EBT bioavailability enhancement were the mucoadhesive nanoparticulate system, self-nanoemulsifying delivery system, fast dissolving tablets, and self-microemulsifying delivery system [5,6,7,8]. Some success has been achieved with self-emulsifying delivery systems but need more efforts to make delivery system simple for commercialization.

The marvelous potential prospective of micelles to facilitate drug delivery to target sites through imminent pharmaceutical excipients increase the chances of improving the bioavailability of poorly soluble drugs. For instance, lipophilic drugs are frequently incorporated in micelles to increase the lymphatic transport of these drugs. Micellar systems have displayed tremendous enactment during permeation at target sites. Interesting, mixed micelles are providing the platform for the oral delivery of poorly soluble drugs [9]. Typically micelle consist of core-shell self-assembled structure of amphiphilic molecules [10]. Micelles improve the drug absorption by modulating the membrane permeability [11]. In addition, micelles size and morphological properties help to increase the absorption through the biological barriers [12]. The different amphiphilic molecules of mixed micelles provide significant benefits in oral drug delivery by improving physical stability to micelles and high drug loading [13]. The solidification of micelles is necessary for stable formulation development [14]. Mixed micelles are intended to incorporate in oral films to avoid expensive lyophilizing or spray drying techniques. Micelles integrated sublingual films provide a whimsical drug delivery system to improve bioavailability of poorly water-soluble drugs [15]. Importantly, drug degradation in the gastrointestinal tract can be avoided by administering through oral mucosal route. The sublingual route has appeared as a substitute to the oral route for immediate drug delivery [16]. As acute allergic reactions need immediate remedies, sublingual route of drug delivery was thus considered appropriate. The presence of micelles in sublingual films would increase the permeation of drug. Therefore, to achieve the desire bioavailability of EBT, the use of micelles could be an economical and significant way to overcome the physiological barriers [17].

The rational selection of amphiphilic molecules can play significant role for development of intended drug delivery system [18]. The reason of selection of poloxamer-188 compared to other high-molecular-poloxamers (HMPs) is that it produces small size micelles and sufficient stability to micelles [19,20]. Further, Poloxamer-188 is less mucoadhesive than HMPs. The small molecular weight of poloxamer-188 helps to release drugs at a prompt rate compared to HMPs [21]. The critical micelle concentration (CMC) 0.040–0.46 mM of poloxamer-188 is reasonable for the development of micellar systems. Poloxamer-188 can re-seal the disruptions of cell membranes, thus maintaining the cell integrity [22]. Additionally, poloxamer-188 displayed low toxicity in comparison with other HMPs in previous studies [23]. The role of poloxamer-188 is well established to increase the solubility of poorly soluble drugs [24]. In addition, poloxamer-188 block copolymer is a commonly used as micellar carrier for the delivery of poorly soluble therapeutics agents. The hydrophilic and hydrophobic portion of copolymer has the ability to self-assemble and to impede P-glycoprotein transporter. For instance, the activity of sildenafil (SIL) was boosted through poloxamer-188 micelles encapsulation by the research team of Charisopon Chunhachaichana [19]. Additionally, poloxamer-188 was frequently used with other polymeric and amphiphilic molecules for the formation of micelles [25,26]. For example, the team of Xin Jin evaluated mixed micelles of poloxamer-188/phospholipid for the delivery of juglone in breast cancer [27]. Furthermore, d-α-tocopheryl polyethylene glycol succinate 1000 (TPGS) is an FDA-approved biological modifier that is used in nano-carrier systems for the delivery of poorly soluble drugs [28]. The TPGS-1000 inhibits the efflux transporters specifically p-glycoprotein, improves oral absorption, and stabilizes the sublingual films [29]. The TPGS-1000 micelles were successfully used for the delivery of curcumin to brain [30,31]. The poloxamer-188/TPGS-1000 mixed micelles have been used as carrier for drug delivery in past [32,33]. The concentration of amphiphilic molecules are kept high above their CMC in this study. The CMC value of TPGS-1000 is about 0.02 mM [32]. The properties of poloxamer-188 and TPGS-1000 such as biocompatibility, safety, high encapsulation efficiency, as well as high solubilization capacity make them suitable candidates that can be used to enhance the bioavailability of poorly soluble drugs [34].

Therefore, the objective of this work was to develop novel poloxamer-188/TPGS-1000 mixed micelles-loaded sublingual films for trans-mucosal delivery of EBT to enhance its bioavailability.

The novelty of this research work is that mixed micelles of EBT was developed first time for the absorption enhancement using poloxamer-188/TPGS-1000. Further uniqueness of this study was the stabilization of EBT encapsulated micelles through integration into sublingual films. The ultimate goal of current formulations was thoroughly characterized to determine the level of improvement in bioavailability of EBT.

## 2. Materials and Methods

### 2.1. Materials

Ebastine (EBT) was purchased from Simz Pharmaceuticals, Lahore, Pakistan. Poloxamer-188 was gifted from BASF, Ludwigshafen, Germany. d-α-tocopheryl polyethylene glycol succinate 1000 (TPGS-1000) was supplied by Antares Health Product Incorporation, Jonesborough, TN, USA. Hydroxypropyl methylcellulose-E5 was generously provided by Colorcon Incorporation, Harleysville, PA, USA. Glycerol was taken from Wimits Pharmaceuticals, Lahore, Pakistan. All other used reagents and chemicals were of analytical grade unless otherwise specified.

### 2.2. Preparation of Micelles

A thin-film hydration method was used to prepare micelles of EBT. Briefly, different proportions of EBT, P-188, and TPGS-1000 were dissolved in ethanol using a round-bottom flask (Table 1). Then, this bottle was attached to a rotatory evaporator (Rotavapor-R-300^®^, Buchi Labortechnik AG, Flawil, Switzerland) and heated at 50 °C under vacuum to obtain a thin film of polymers. Later, a small quantity of distilled water (10 mL) was added to hydrate the film following magnetic stirring using 200 rpm speed at 50 °C for 1 h to form micelles. The EBT (10 mg) was incorporated in all micelles formulations [35].

### 2.3. Preparation of EBT Micelles-Loaded Orodispersible Sublingual Films

**Figure 1:**
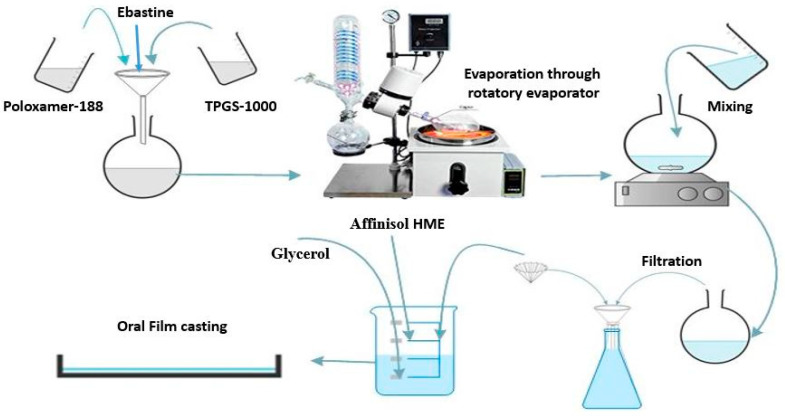
Schematic diagram of the manufacturing process of micelles-loaded orodispersible sublingual films.

Hydroxypropyl methylcellulose (HPMC-E5, methoxyl content 28–30 wt%, and hydroxypropoxyl content 7–12 wt%) having molecular weight 28700 Da was used as a film-forming polymer to prepare orodispersible films by means of a simple solvent casting method (Figure 1). For that purpose, HPMC solutions were prepared in different concentrations (see Table 2) and left overnight to remove bubbles. Then, the optimized MTP-4 micelles dispersion (mass concentration ratio of TPGS-1000 to poloxamer-188; 4:1) containing total concentration 200 mg/10 mL of EBT was added into the HPMC (10–15%) solution under continuous stirring [36]. Afterward, remaining excipients including glycerol (3–4%), crospovidone (2%), and polysucralose (1%) were added stepwise in the film dispersion. The final volume was made 20 mL with double distilled water. The final mass concentration of EBT in film solution was kept at 10 mg/mL. Subsequently, the prepared solutions with viscosity of approximately 6350 mPa.s were poured into glass petri dishes and inverted funnels were placed on petri dishes for smooth drying and subsequently put in an oven at 35 °C. The obtained films (see the Supplementary Materials, Figure S1) were cut in 2 × 2 cm^2^ sizes and stored in a desiccator after wrapping with aluminum foil for further use. Similarly, orodispersible sublingual films (OSFs) of pure drugs (without mixed micelles) were also prepared to evaluate effect on bioavailability.

### 2.4. Characterization of Mixed Micelles

#### 2.4.1. Particle Size, Polydispersity Index (PI), and Zeta Potential

The size, charge, and PI of micelles were tested using a Zeta sizer (Malvern Zetasizer 3000HS, Malvern Instruments, Malvern, UK). The average results were assessed from triplicate values [37].

#### 2.4.2. Drug Loading and Entrapment Efficiency (EE%)

A weigh amount of micelles was dissolved in methanol in a volumetric flask. Then the mixture was centrifuged to obtain drug solution and volume of flask was made 100 mL with methanol. The second dilution was made with phosphate buffer pH 6.8 to 100 mL. The assay was performed on a UV–VIS spectrophotometer (UV 2600, Shimadzu, Japan) using 252 nm wavelength [37]. The entrapment efficiency was calculated by following formula:

(1) $$\mathrm{Entrapment}\;\mathrm{efficiency}\;\left(\%\right)\;=\frac{\mathrm{Practical}\;\mathrm{drug}\;\mathrm{obtained}\;\mathrm{in}\;\mathrm{micelles}\;}{\mathrm{Theoretical}\;\mathrm{drug}\;\mathrm{added}\;}\;\times \;100$$

(2) $$\mathrm{DL}\;\left(\%\right)=\frac{\mathrm{Amount}\;\mathrm{of}\;\mathrm{drug}\;\mathrm{encapsulated}\;\mathrm{in}\;\mathrm{micelles}}{\mathrm{Total}\;\mathrm{weight}\;\mathrm{of}\;\mathrm{micelles}}\;\times \;100$$

### 2.5. Characterization of Micelle Loaded Orodispersible Sublingual Films

#### 2.5.1. Weight, Thickness, Disintegration Time (DT), Surface pH, and Content Uniformity

Films were weighed using an analytical balance (Sartorius, model Secura125-1S, Aubagne, France) with reliability of 0.01 mg to 120 g [38]. A digital micrometer (Mitutoyo Corporation, Kawasaki, Kanagawa, Japan) was used to measure the thickness of films from three different points and the average was calculated [39]. A petri dish technique was used to figure out the time required for disintegration of a film. In a petri dish of 6.5 cm diameter, 2 mL of simulated salivary fluid of pH 6.8 was poured. Then a film was placed in this petri dish at 37 °C ± 2 °C and shaken at 50 rpm using a horizontal orbital shaker to note the disintegration time [40]. The dose size per unit area was calculated by cutting the film into pieces and dissolving in 50 mL methanol individually. Then, further dilutions were made using phosphate buffer pH 6.8. Afterwards, the EBT contents per unit area was measured using a UV–VIS spectrophotometer (UV-2600 spectrophotometer, Shimadzu, Kyoto, Japan) at 252 nm wavelength.

#### 2.5.2. Folding Endurance of Film

The oral films were folded repetitively in the same direction and the numbers of folds were counted. The number of folds without breaking provided the endurance value of each film.

#### 2.5.3. Tensile Strength Measurement

A film strip of 2 × 2 cm^2^ size was attached to a static pan from one end while other end was attached with a hanging pan. The weight required to break the film was gradually increased on hanging pan. The weight with which the film was ruptured was taken as a breaking force. The tensile strength of film was calculated by using following formula:

(3) Tensile strength (Kgcm2)=Breaking Force (Kg)Area of Film(cm2)

#### 2.5.4. Water Uptake/Loss Studies of Films

The pre-weighed films were placed in petri dishes containing 15 mL of distilled water and incubated for 30 s at 30 °C. Subsequently, the swollen films were weighed after incubation period. The initial weight of film were subtracted from final weight and then divided by initial weight to determine the swelling index of films [41]. Similarly, the loss on drying of OSFs was determined through moisture analyzer (Sartorius-MA-100, Sartorius AG, Gottingen, Germany) keeping the temperature at 105 °C in automatic mode [42].

(4) $$\mathrm{Swelling}\;\mathrm{index}\;=\;\frac{{(W}_{i}{\;-\;W}_{f})}{W_{i}}\;\times \;100$$

where (W_i_) is initial weight and (W_f_) is final weight of films.

#### 2.5.5. Reconstitution of the OSFs Containing EBT-MTP Micelles

The OSFs integrated with EBT-MTP micelles were dispersed in water to obtain micelles. The OSFs were reconstituted with distilled water through gentle stirring for 10 min. The obtained micelles solutions were evaluated for particle size, and entrapment efficiency [15,43]. Further, particle count was determined to confirm micelles formation by using a liquid particle counter (Pamas-S50, Pamas GmbH, Rutesheim, Germany).

#### 2.5.6. Scanning Electron Microscopy (SEM)

A scanning electron microscope (Hitachi S-4700, Hitachi High-tech Corporation, Tokyo, Japan) was used to study the morphology of pure drug and of the developed micelles-loaded films. The films were gold coated by sputter coater (Hitachi MC-1000 Ion Sputter Coater, Hitachi High-tech Corporation, Tokyo, Japan) and scanned.

#### 2.5.7. Atomic Force Microscopy (AFM)

The morphology, diameter, and roughness of the oral film were determined by using atomic force microscopy (Bruker-Dimension-XR, Bruker Corporation, Billerica, MA, USA). The AFM images of samples were collected by running machine in tapping mode with a tapping probe at scanning rate of 0.9 Hz [44].

#### 2.5.8. Fourier Transform Infrared Spectroscopy (FTIR)

The possibility of interaction in excipient or with the drug was studied with the help of FTIR studies. The IR spectra of pure drug, polymers, physical mixtures, and of formulated films were scanned between 400 and 4000 cm^−1^ on Agilent Cary 360 (Agilent Scientific Instruments, Santa Clara, CA, USA) equipped with diamond ATR at resolution of 4 cm^−1^.

#### 2.5.9. Differential Scanning Calorimetry (DSC)

Differential scanning calorimetry scans were recorded by using a diffractometer (SDT-650, TA instruments, New Castle, DE, USA). The aluminum pans were used for reference and samples. The samples were scanned at 20 K/min from 30 to 400 °C using nitrogen purging rate of 20 mL/min to evaluate thermal behavior of drug, polymers, and films.

#### 2.5.10. Thermogravimetric Analysis (TGA)

The changes in physical and chemical properties of pure drug and micelle loaded orodispersible films were determined by performing thermogravimetric analysis. The analysis was performed by running the SDT-650 (TA instruments, New Castle, DE, USA) at 10 °C/min from 30 to 400 °C under nitrogen purge.

#### 2.5.11. X-ray Diffraction (XRD)

The powder X-ray diffraction (XRD) spectra of drug, polymers, and films were obtained between 5°~70° angular range of 2θ using a XRD machine (D8 Advance, Bruker Corporation, Billerica, MA, USA). The samples were run by setting PXRD machine at voltage of 30 kV, current 20 mA, and angular speed of 2θ/min.

#### 2.5.12. In Vitro Drug Release

In vitro drug release of the pure drug and films was carried out using USP paddle dissolution apparatus (Biobase Biodustry, Jinan, China). The film size was cut per unit dose and placed in a steel wire mesh. The mesh along film was tied with paddles and hanged in dissolution vessel containing 900 mL of phosphate buffer pH 6.8. The dissolution studies were performed using a stirring speed of 50 rpm and the temperature of water bath was maintained at 37 ± 0.5 °C. A 5 mL sample was withdrawn at predetermined time intervals of 5, 10, 20, 30, 40, 50, and 60 min was replaced with fresh medium. The samples were filtered, diluted, and analyzed at 252 nm wavelength using a UV–VIS spectrophotometer (UV-2600 Shimadzu, Kyoto, Japan). All samples were in triplicate [45,46].

#### 2.5.13. Pharmacokinetics Study in Rats

Adult male Wistar rats, weighing about 200–220 g with age ranging from 6–8 weeks were purchased from The University of Lahore (UOL), Pakistan. The approval for animal experiments was taken by the Institutional Review Committee, Government College University Faisalabad. (Ref No. GCUF/ERC/2068, Study No. 19668, IRB No. 668, 5 September 2019). The international and institutional guidelines were strictly followed for animal care and ethics. All animals were housed at controlled environment (25 ± 1 °C and 60 ± 10% relative humidity) and 12 h light/dark cycles. All animals were given full access to standard food and water. The rats were divided into two groups with each containing six (*n* = 6). The rats were restricted from food for 12 h (fasting condition) and free access to water was given to both groups. The ether anesthesia was given to all animals to place films in oral cavity. Pure EBT 10 mg/kg and EBT-micelles-films equivalent to 10 mg of EBT were administered to the rats. The pure drug was suspended in 1 mL of water and given to rat through an oral gavage. The OSF was cut into 0.4 × 0.4 cm^2^ pieces, and each piece containing 2 mg of EBT was placed under rat’s tongue using spatula and forceps. Blood samples were taken in heparinized tubes from orbit vein at predetermined time intervals (0.5, 1, 2, 3, 4, 5, 6, 8, 10 and 24 h). The samples were centrifuged at 8000 rpm for 10 min to obtain plasma and stored at −20 °C for further analysis [36]. The drug was extracted by centrifuging mixture of 200 μL plasma and 400 μL absolute methanol at 8000 rpm for 5 min at 4 °C. The residue was dried in another tube at −40 °C under nitrogen stream. The reconstitution of residue was done with methanol. The suspension was vortexed and centrifuged at 8000 rpm and supernatant was collected for further analysis using HPLC [46]. The isocratic reverse phase HPLC (10 AT, SPD 10A, Shimadzu, Japan) equipped with C-18 column was used to analyze the drug using acetonitrile: Ammonium acetate mobile phase at 257 nm wavelength with a flow rate of 1 mL/min.

#### 2.5.14. Toxicological Studies in Rats

The Organization for Economic Co-operation and Development (OECD) guidelines were strictly followed for the performance of toxicity study in animals. The toxicity study protocols were approved by Institutional Ethical Review Committee of Government College University Faisalabad (Ref. No: GCUF/ERC/2068, 5 September 2019). Twelve healthy rats were divided into two groups (each group with 6 rats) for testing purpose. Controlled group A was treated with adequate food and water while Group B was treated with developed film containing EBT equivalent to 10 mg/kg body weight. The food consumption pattern, physical appearance, and mortality rate were monitored on a daily basis whereas the body weight was checked on a weekly basis. After 14 days, blood samples were withdrawn for the evaluation of biochemical and hematological parameters.

#### 2.5.15. Cells Viability Assay

A cell viability test was performed by culturing human buccal epithelium cell line (TR146, Merck Pakistan authorized distributor of Sigma-Aldrich) in 96-well plates with a density of 1 × 10^4^ per well and incubated at 37 °C for 24 h. The prepared cells were incubated with EBT and FC-5 oral film at 37 °C for 24 h. The cells were washed with phosphate buffer and shifted to fresh medium after every incubation. The supply of 95% fresh air with 5% CO_2_ was maintained during the entire incubation period. The 20 µL MTT (5 mg/mL stock) solution was added in each well and incubated for next 2 h. The assay was carried out with optical density at 570 nm using microplate reader (EnSight^®^, PerkinElmer, Waltham, MA, USA).

(5) $$\mathrm{Cell}\;\mathrm{viability}\;(\%)\;=\;\frac{\mathrm{Absorption}\;\mathrm{of}\;\mathrm{test}\;\mathrm{cell}}{\mathrm{Absorption}\;\mathrm{of}\;\mathrm{control}\;\mathrm{cell}}\;\times \;100$$

### 2.6. Statistical Analysis

The data were detailed as mean ± standard deviation (SD). One-way ANOVA was performed for statistical analysis using SPSS software (version 16.0, SPSS incorporation, Chicago, IL, USA). *p*-values less than 0.05 were considered to be statistically significant.

## 3. Results

### 3.1. Particle Size, Polydispersity, Zeta Potential, and Entrapment Efficiency of Micelles

**Figure 2:**
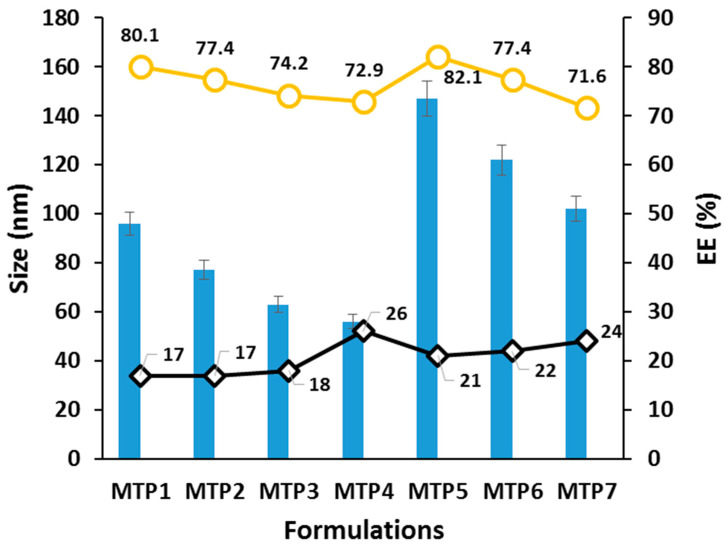
Particle size, polydispersity index, zeta potential, and entrapment efficiency (EE) (%) of micelles formulations.

The average size of EBT-loaded mixed micelles of poloxamer-188 and TPGS-1000 was 73 ± 2 nm while the average PI value was 0.15 ± 0.024. The particle size after the dispersion of orodispersible film was 83 ± 2 nm with a PI value of 0.19 ± 0.032 showing a narrow size distribution. A slight increase in the size of particles was observed after re-dispersion of film. The average surface charge of micelles was −20.7 ± 3 mV. The entrapment efficiency was high in the case of MTP5, MTP6, and MTP7 micelle formulations. Above CMC value, the EE% was increased with increase of micelles forming components (TPGS-1000/poloxemer-188). Drug loading values were found in range of 4.8 to 10.61% ± 2%. It is clear from the results that the size of micelle was not significantly changed upon incorporation in films as shown in Figure 2.

### 3.2. Physical Properties of Orodispersible Sublingual Films

The average thickness of films was 0.158 ± 5 mm while the average weight was 70.04 ± 1.3 mg. The pH of all film surfaces was about 6.8. The prepared films were disintegrated within few seconds, however the thicker films showed comparatively higher disintegration time (DT). The DT of the films was not influenced due to their high mechanical properties. The average contents of EBT in each 2 × 2 cm^2^ strip was around 100.9 ± 5%, which was within pharmacopoeia limits. The loss on drying was found between 0.06 to 1.59% ± 0.03%. The higher folding endurance values confirmed that the films were appropriate for storage and transportation. Nevertheless, the folding endurance and mechanical properties of the films were affected as a function of concentration of film former and plasticizer (Table 3). The lowest disintegration time (28 s) was presented by FC-5 film. Similarly, the folding endurance and tensile strength were found to be 34 and 1.82 kg/cm^2^, which were higher compared to other formulations. The swelling index of FC-5 was reasonably higher than FC-1 and FC-3 formulations. Based on the disintegration time, folding endurance, and tensile strength of films, FC-5 was selected for further studies like SEM, FTIR, XRD, TGA, DSC, AFM, PK, and toxicity.

### 3.3. Reconstitution of OSFs Containing EBT-MTP Micelles

The confirmation of micelles formation after reconstitution of OSFs with water was mainly carried out through particle size and entrapment efficiency analysis. The particle size of the micelles was found smaller after reconstitution compared to their original sizes. The size of particles was found in the range of 50.2 to 59 nm. After reconstitution, colloidal solution was easily formed from OSFs and particles-count was found more than their original numbers. In addition, re-formation of micelles was established by the entrapment efficiency of micelles which was not affected upon incorporation in film. The obtained entrapment efficiency was in the range of 70 to 80% after reconstitution. The particles count was found 3 times higher than original count after re-dispersion. The comparative results of particle size and entrapment efficiency of micelles before and after the reconstitution are similar regardless of small variation.

### 3.4. Scanning Electron Microscopy (SEM)

**Figure 3:**
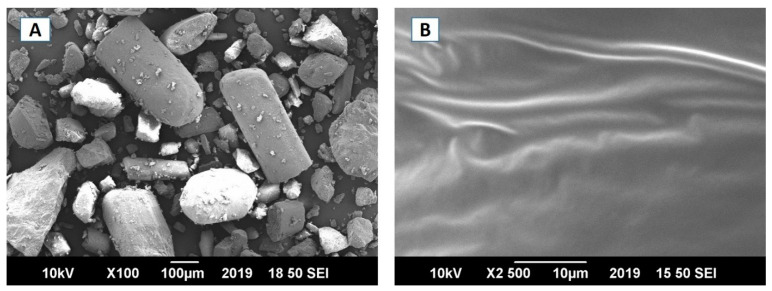
SEM images of (**A**) EBT, (**B**) FC-5 formulation of Orodispersible sublingual film.

The micrographs of pure EBT (panel A) and micelles-loaded film FC-5 (panel B) are presented in Figure 3. The irregular crystals of EBT could be clearly seen in panel A. The length of the crystals differed a lot as the smaller crystals were observed to be adhered to the larger crystals. In comparison, the micelles containing films showed overall smooth texture in the SEM images (panel B)**.** Importantly, amorphous characteristics were reflected in the SEM micrographs due to the lack of initial crystal shape of EBT in oral film.

### 3.5. Fourier Transform Infrared Spectroscopy (FTIR)

**Figure 4:**
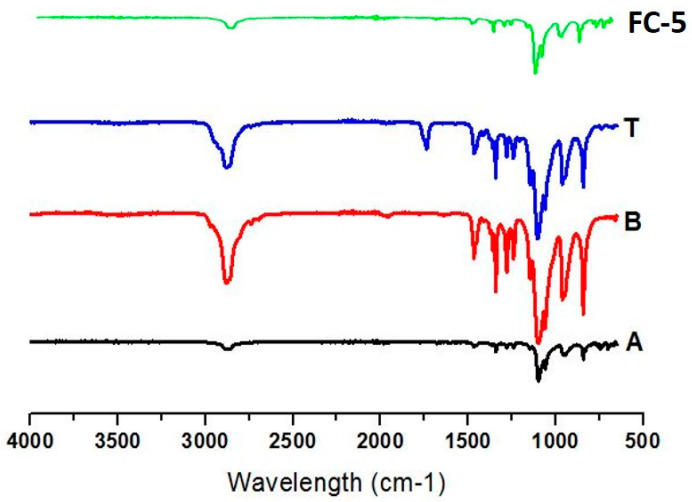
FTIR spectra of (A) pure EBT, (B) poloxamer-188, (T) TPGS1000, and (FC-5) micelles-loaded film.

The pure EBT presented characteristic peaks of C=O stretch at 1672 cm^−1^, C=C stretch at 1452 cm^−1^, and C–N stretch at 1262 cm^−1^, respectively. The IR spectrum of pure drug with reference to their physical mixture and films showed no noticeable incompatibility among the ingredients of formulation and drug. No major shift was observed in the spectra of micelle loaded orodispersible film as shown in Figure 4. The significant similarities were found in functional groups peaks of pure EBT and FC-5 film formulation. The carbonyl functional group appeared at 1672 cm^−1^ in FC-5 indicated the existence of EBT in the oral film.

### 3.6. X-ray Diffraction (XRD)

**Figure 5:**
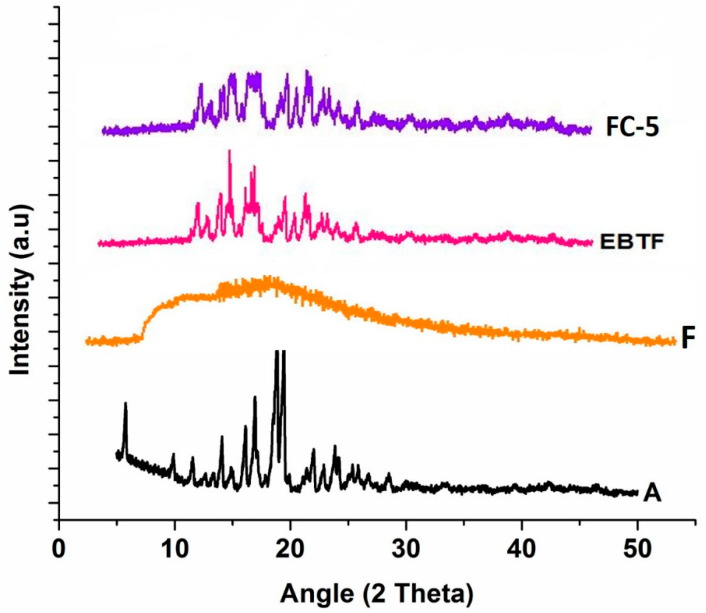
XRD diffractograms (A) EBT drug, (F) blank film, (EBTF) EBT plain film, (FC-5) micelles-loaded film.

The crystallinity of EBT and micelles was assessed by X-ray diffractograms as shown in Figure 5. The characteristic diffraction peaks of EBT appeared between 17.26° and 19.53° confirming its crystalline structure. The diffractogram of HPMC did not reveal large crystalline peaks reflecting its amorphous nature. XRD pattern of EBT-loaded plain films exhibited crystalline peaks at 17.13° and 19.31°, however a decrease in the number of crystalline peaks was found in the plain film, but EBT crystalline peaks did not disappear entirely. In contrast, peaks were diminished in orodispersible film indicating reasonable conversion of crystalline drug into amorphous form.

### 3.7. Thermogravimetric Analysis (TGA)

**Figure 6:**
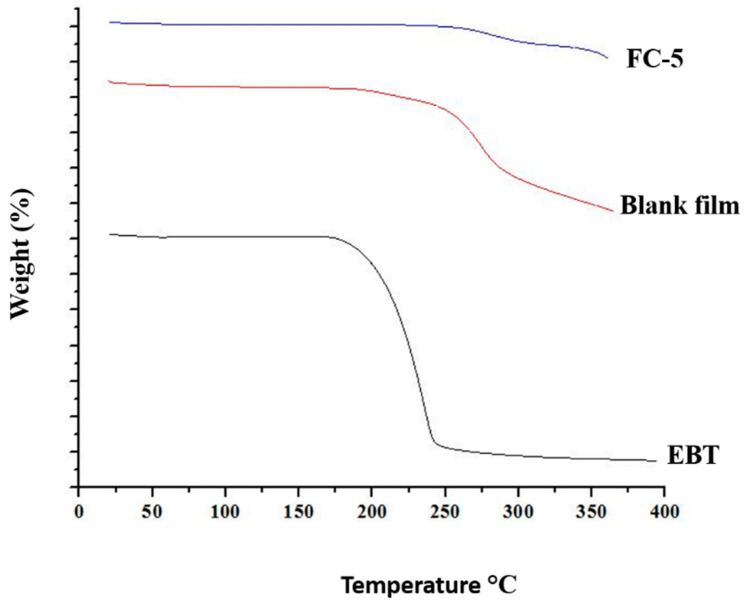
Thermograms of pure EBT, blank film, and EBT micelles-loaded orodispersible sublingual film.

The thermograms of pure drug, blank, and EBT micelles-loaded film were performed to check weight changes for monitoring their thermal stability. The thermograms of pure drug and films depicted elimination of water up to 150 °C. As the temperature raised above 150 °C, the decomposition of ingredients was observed (Figure 6). The thermal decomposition of EBT was evident from the TGA thermogram showing a decomposed temperature of EBT between 210.6 °C and 240.3 °C. Total decomposition of the EBT took place around 400 °C. In comparison, the overall decomposition of EBT in plain film and FC-5 orodispersible sublingual film was not more than 50%. This was indicative of increased thermal stability of EBT in oral films compared to pure EBT.

### 3.8. Differential Scanning Calorimetry (DSC)

**Figure 7:**
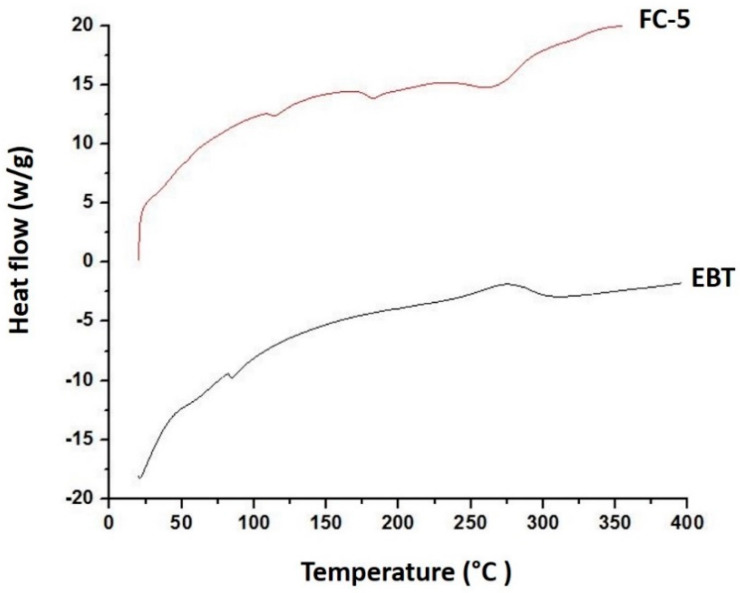
DSC curves of pure EBT and micelles-loaded orodispersible sublingual film (FC-5).

The physicochemical interaction and thermal behavior of drug and excipients were further evaluated using DSC. The possible interaction among ingredients could be monitored by the change of endothermic or exothermic peaks. The EBT exhibited sharp melting endothermic peak at 86 °C, which were not present in FC-5 formulation depicting its conversion into amorphous form (Figure 7). The DSC curve of FC-5 seemed to be smooth showing decreased crystallinity and possibly increased TPGS-1000 and poloxamer-188 homogeneity. A slight endothermic plateau near 180 °C could be due to the melting temperature of the HPMC.

### 3.9. Atomic Force Microscopy (AFM)

**Figure 8:**
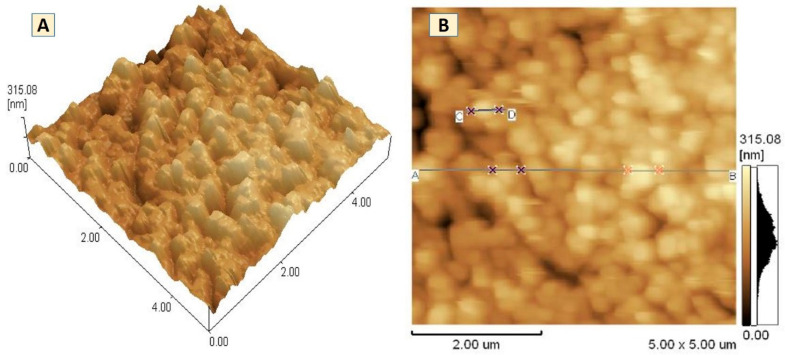
Atomic force microscopic images of FC-5 formulation (**A**) 3D and (**B**) 2D images.

The AFM microscopic images uncovered morphology, average height, roughness, and diameter of the prepared orodispersible films (Figure 8). The height of the micelles-loaded film was found near 315.08 ± 6.4 nm. No increase in height of film confirmed the small size of micelles with maintaining their shape and integrity after loading in the film. However, a very small variation in height was noticed at three different point (two points at A-B, and one point at C-D) (Figure 8B). Figure 8A showed the penetration pattern of micelles in the film.

### 3.10. In Vitro Dissolution Studies

**Figure 9:**
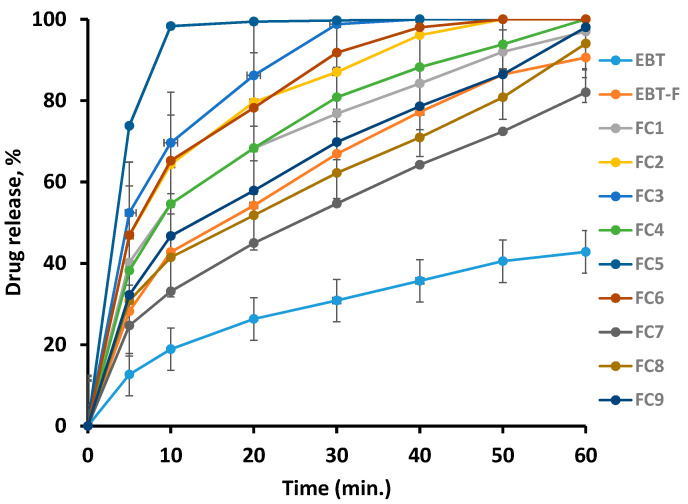
In vitro drug release profile from pure EBT, plain drug-loaded film, and micelles-loaded orodispersible films.

In vitro release profile of pure drug, EBT-loaded plain, and micelles-loaded orodispersible films (FC1 to FC9) is shown in Figure 9. The percentage of drug releases within the first 30 min from oral films (EBT-F, FC-1, FC-2, FC-3, FC-4, FC-5, FC-6, FC-7, FC-8, FC-9) was 66.89%, 76.8%, 87.0%, 98.8%, 80.8%, 100.0%, 91.8%, 54.7%, 62.2%, and 69.8%, respectively. In contrast, pure EBT exhibited a distinct slower release about 30.86% within 30 min (*p* < 0.05). Furthermore, the drug from micelles-loaded film (FC-5) was released above 70% within 5 min, whereas only 28.2% of the drug was dissolved in 5 min from thee plain EBT film (EBT-F) (*p* < 0.05).

### 3.11. Pharmacokinetic Parameters in Rats

**Figure 10:**
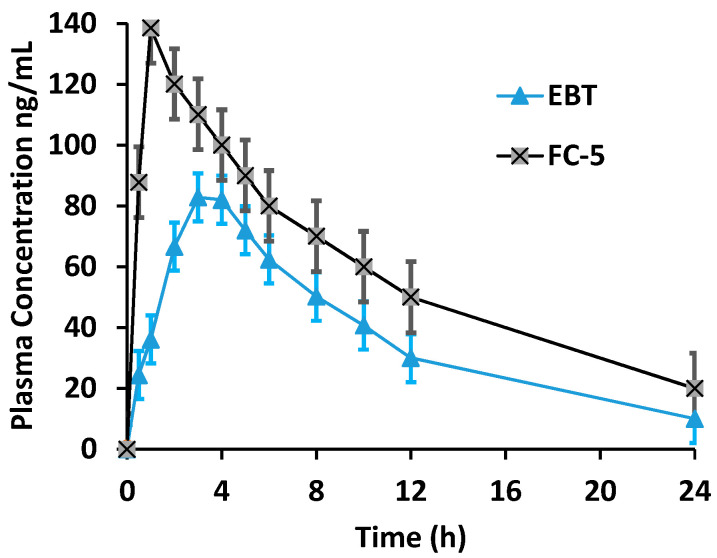
Mean plasma concentration-time curve of pure EBT and FC-5 orodispersible film after oral administration in rat (*n* = 6).

The pharmacokinetic profile of optimized formulation (FC-5) was determined and compared with pure drug as shown in Table 4. The maximum plasma concentration was obtained from FC-5 film at 2.5 h relative to pure EBT achieving C_max_ at 4 h (*p* < 0.05) (Figure 10). In addition, the area under curve (AUC) of FC-5 was increased 2.18 fold compared to the pure drug (*p* < 0.05).

### 3.12. Toxicological Studies

The general physical conditions of rats such as body weight, water and food intake, common signs of illness, salvation, dermal and ocular irritation were observed daily while the body weight was recorded on the 1st, 7th, and 14th day of treatment. The pattern of food intake was found to be consistent in both groups. No mortality was seen until the end of toxicity study. A slight variation in weight of rats was observed for tested group in comparison to the control group, however, it was less than statistical significant (*p* < 0.05) (Table 5). In contrast, the hematological and biochemical results showed statistical insignificant (*p* < 0.05) for control (Group A) as well as for the tested group (Group B) (See Table 6 and Table 7). A small reduction in mean platelet count was observed in the tested group but no noticeable difference was seen in hematological and biochemical data of the control and tested groups. The rats showed good tolerance to drug concentration with no abnormality signs even after 14 days.

### 3.13. Cell Viability Assay

**Figure 11:**
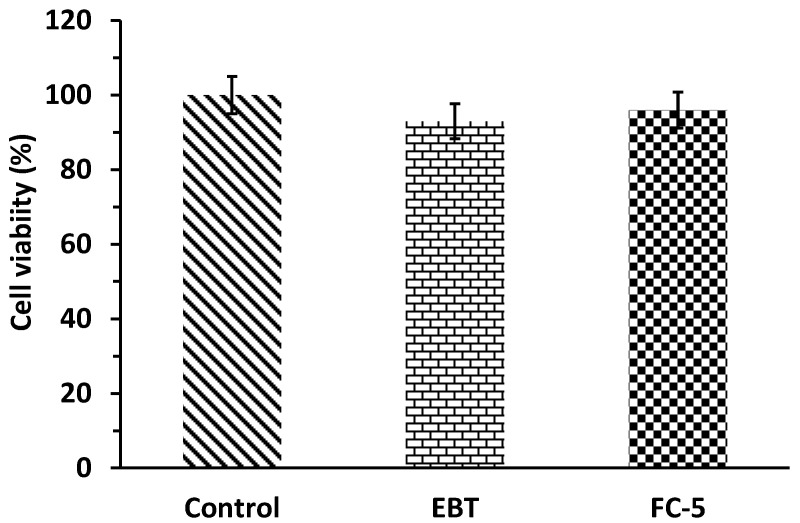
Cell viability of human buccal epithelium cell lines (TR146) after exposure to pure EBT and FC-5 micelle loaded films.

The results of MTT assay (3-[4, 5-dimethylthiazol-2-yl]-2, 5 diphenyl tetrazolium bromide) are presented in Figure 11. The concentration of 100 mg/kg of EBT and FC-5 orodispersible film presented 93% and 96% cell viability compared to control cells (Figure 11). In the cells, the succinate dehydrogenase enzyme converted the tetrazolium salts into the formazan precipitate, which suggested the cell viability percentage. For this, formazan precipitate was dissolved with dimethyl sulfoxide (DMSO) and quantified UV-spectrophotometrically. The above 90% of cell viability for both EBT and FC-5 formulation clearly indicated their non-toxic behavior to cells. Thus, these finding confirmed the biocompatibility of FC-5 orodispersible films.

## 4. Discussion

The high drug loading and entrapment efficiency are attributed to the nature of micelles components. The drug was entrapped in the lipophilic core of micelles and covered by hydrophilic head group [37]. The mass concentration ratio of TPGS-1000 and poloxamer-188 revealed significant effects on entrapment efficiency of micelles. The mass concentration ratio effects were evaluated by changing concentration of TPGS-1000 and poloxamer-188. When concentration of TPGS-1000 was increased by keeping poloxamer-188 concentration constant, significant improvement in entrapment efficiency (EE) of micelles was observed. Contrarily, %EE was decreased by increasing the poloxamer-188 concentration and keeping TPGS-1000 concentration constant. Thus further studies were carried out with 4:1 of TPGS-1000/poloxamer-188. Additionally, the total concentration of TPGS-1000/poloxamer-188 showed influence on EBT entrapment efficiency as well. The increase of total concentration of TPGS-1000/poloxamer-188 from 50 mg to 200 mg improved the entrapment efficiency remarkably. The 10 mg/mL concentration of EBT provided stable and small-sized micelles. The solubility of EBT was increased due to the integration into hydrophobic core of micelles [19,31].

The mixed micelles incorporated orodispersible sublingual films provided strikingly superior results compared to pure drug and plain-EBT-loaded films. It was noticed that the concentrations of film-forming polymers (HPMC) and plasticizer (glycerol) played a vital role in the physical appearance and mechanical strength of the films. HPMC was selected as film former based on the adequate mechanical strength and fast disintegrating properties [47]. Clearly, the glycerol containing films were smooth, translucent, and flexible [48]. Folding endurance increased as a result of increasing plasticizer concentration in the prepared films [49]. The high concentration of a film-forming a polymer without the inclusion of a plasticizer resulted in brittle film [47]. Thus, the proper selection of film-forming polymer and plasticizer concentration is necessary for appropriate structural integrity and folding endurance of film [50]. The mechanical properties of films were improved prominently with increasing concentration of HPMC and glycerol. Evidently, the presence of micelles in the film did not affect the characteristics of the film [51]. When micelles were loaded in the films it was observed that the micelles were completely covered by the higher polymer contents. The findings revealed that the stability of micelles was improved by incorporating micelles into the film [52]. The optimized formulation FC-5 exhibited good folding endurance and mechanical properties [53]. Furthermore, the presence of micelles in formulation increased the mechanical properties of films [54]. Therefore, it was identified that the concentration of micelles in film had an impact on elasticity, disintegration behavior, and mechanical properties of film. So, optimum particle loading in films is essential for reasonable results [54,55]. The optimized film was selected based on disintegration time (DT), mechanical properties, thickness, average weight, softness, flexibility, and translucence. The physicochemical properties of the FC-5 film presented ideal properties for the prompt delivery of EBT. The film FC-5 completely disintegrated within 28 s. The disintegration time of less than 180s is considered suitable for fast disintegrating films [55,56]. The OSFs showed reasonable swelling index for fast disintegration of films. The HPMC usually shows swelling about 10 to 12% in aqueous media [57]. The presence of superdisintegrant (crospovidone) might be the reason of higher swelling of FC-5 [57,58,59]. The folding endurance was achieved equitably high with HPMC. This could be credited to the presence of plasticizer (glycerol) in oral films [36,38]. The tensile strength of developed-OSFs was almost matched with tensile strength of buccal films prepared by Dalia Abouhussein et al. [58]. The higher tensile strength of films was due to appropriate proportion of HPMC and glycerol in the sublingual films. Additionally, higher concentration of HPMC improved the mechanical strength to films [60,61]. After reconstitution of OSFs with water, the entrapment efficiency of micelles remained intact [62]. Similarly, the micelles’ size was not increased upon incorporation in oral films. The total particle count was increased upon reconstitution of OSFs, which was also an indication of micelles formation. In addition, the micelles’ size was detected below their original sizes. This increase in entrapment efficiency and particle count and decrease in particle size can be attributed to micelles. The mixed micelles of TPGS/poloxamer-188 were formed above their CMC [19]. The results suggested that micelles integrated sublingual films were successfully prepared with desired entrapment efficiency and particle size. The FC-5 presented ideal properties for the transportation of EBT-micelles through sublingual route.

The FTIR spectra provided clear and prominent peaks at same wave numbers. The decrease in peak size may be correlated with the reduced stretching and bending motions within film polymers. No PXRD peak with large intensity was observed in films, which was an indication of conversion of EBT crystalline structure into amorphous state. The films containing micelles did not show crystalline diffraction peaks. However, the low-intensity peaks were observed in few films. The conversion of crystalline state of EBT into amorphous form may possibly be attributed to the inclusion of TPGS-1000 and poloxamer-188 in formulations [63]. Moreover, the x-ray diffraction depicted that the changed internal structure of drug crystals might be the reason for enhancing the solubility, dissolution rate and bioavailability of EBT. Additionally, the sharp peak of EBT was completely suppressed in the DSC thermal curve of FC-5 which also confirmed the XRD results. In the micelles-loaded film, no endothermic peak of EBT was observed confirming its conversion into amorphous form. The weight loss of EBT during thermogravimetric analysis was attributed to structural degradation with temperature. The oral films revealed thermal degradation above 250 °C reflecting improved thermal stability of EBT after incorporation in films. The smooth surface and uniform distribution of micelles in the films was evidenced from the AFM data. Furthermore, the AFM findings indicated that the particle size of embedded micelles was closer to the results obtained from dynamic light scattering. A variation in the height of micelles could possibly be the reason for minor rough surface of oral film. The height of the film was not distorted with the inclusion of micelles in film. Along with small height, the broad surface area of film contributed to the rapid dissolution of micelles in the oral cavity, thereby facilitating greater absorption of EBT [64]. The disintegration and dissolution behavior of micelles-loaded films presented prompt and better results as compared to the pure drug. The sedimentation of pure drug was visually observed in media vessels. The developed orodispersible plain films were disintegrated smoothly whereas the film containing micelles were dispersed even quickly. A rise in the dissolution rate of EBT may be due to the combined efficiency of micelles-orodispersible film including the conversion of crystalline structure to amorphous form, reduced particle size, and large surface area [65].

The pharmacokinetic parameters of EBT oral suspension and micelles-loaded films provided absorption pattern of EBT. The effect of formulations on drug absorption was described by corresponding pharmacokinetic parameters including T_max_, C_max_, and AUC. The highest increase was seen in C_max_ of micelles containing film (FC-5) compared to the pure drug (*p* < 0.05). T_max_ of micelles containing film (FC-5) was much less in comparison with pure drug (*p* < 0.05). Further, the AUC_0–24_ h was 2.20 times greater when compared to the pure drug (*p* < 0.05). The absolute bioavailability of EBT was increased 2.18 fold through OSFs. It is clear from the pharmacokinetic results that micelles-integrated films have more absorptive properties than pure EBT. The OSFs have large surface area and thus disintegrate quickly in the oral cavity in the presence of salvia. The released micelles from OSFs might have increased the permeability across the sublingual mucosal layers [66]. Moreover, small micelles size offers large surface area for drug absorption that could be reason for fast permeability [67,68]. Owing to the fast absorption of micelles from the mucosal cavity and the prevention of first-pass hepatic degradation, the bioavailability of micelles-loaded films was enhanced [44,69]. This may also be attributed to the biopharmaceutical effects of poloxamer-188 as well as modulating drug transporter (P.gp) activities of TPGS-1000 [70]. As expressions of P.gp are less in the oral cavity compared to GIT, so there are minimum chances of receptor manipulation by TPGS-1000 [71]. While maximum possibility of bioavailability enhancement is through absorption mechanism from oral cavity via micelles. The micellar activity of TPGS-1000/poloxamer-188 increased the oral bioavailability many folds. The maximum concentration (C_max_) of EBT was achieved within 1 h after administration. This could be linked to fast absorption of small micelles composed of TPGS/poloxamer. Further, TPGS is also known for enhancing the absorption of lipophilic drugs. The area under curve (AUC) was almost 2.18 times higher compared to pure EBT. The pharmacokinetic data clearly presented that micelles improved the absorption compared to pure EBT. Therefore, mixed micelles containing TPGS-1000/poloxamer-188 could be a suitable vehicle for the transport of poorly water soluble molecules through sublingual route by enhancing transmembrane absorption. In addition, the results of cell viability of developed film (FC-5) showed a safe toxicity profile and good biocompatibility as the biochemical and hematological parameters were not affected. The metabolically active cells were confirmed by the MTT assay through enzyme activity [72]. The results of biocompatibility of FC-5 are in compliance with previously reported studies [73].

## 5. Conclusions

The poloxamer-188 and d-α-tocopheryl polyethylene glycol succinate (TPGS-1000) mixed micelles were successfully loaded into orodispersible sublingual films. Orodispersible film formulation (FC-5) presented the most appropriate results for thickness, weight, surface pH, disintegration time, and mechanical strength. The mixed micelles-loaded orodispersible sublingual films significantly enhanced dissolution of EBT by reducing its particle size, converting into amorphous form, and providing a large surface area. The in vitro and in vivo studies revealed rapid absorption, high permeability, and bioavailability of EBT in the presence of poloxamer-188 and TPGS-1000. The general behavior, body weight variability, hematological, biochemical, and MTT studies showed no significant detrimental effects on the functioning of different vital organs, and thus, the FC-5 mixed micelles-loaded film was considered to be non-toxic for biological system. In conclusion, the developed mixed micelles-loaded sublingual films improved the bioavailability of ebastine significantly, and thus could be an effective delivery system for improving the bioavailability of poorly soluble drugs.

## Figures and Tables

**Table 1:** Composition of micelles formulations.

Formulation Code	Ebastine (mg)	TPGS 1000 (mg)	Poloxamer-188 (mg)
MTP-1	10	50	50
MTP-2	10	100	50
MTP-3	10	150	50
MTP-4	10	200	50
MTP-5	10	50	200
MTP-6	10	50	150
MTP-7	10	50	100

**Table 2:** Composition of micelle loaded orodispersible sublingual films of ebastine.

Formulation Code	HPMC-E5 (%)	Glycerol (%)
FC-1	10	3
FC-2	10	3.5
FC-3	10	4
FC-4	12.5	3
FC-5	12.5	3.5
FC-6	12.5	4
FC-7	15	3
FC-8	15	3.5
FC-9	15	4

**Table 3:** Physical properties of developed orodispersible sublingual films.

Formula Code	Thickness (mm), m.v ± s.d.	Weight (mg), m.v ± s.d.	Disintegration Time (s), m.v ± s.d.	Folding Endurance (*n*), m.v ± s.d.	Tensile Strength (kg/cm^2^), m.v ± s.d.	Swelling (%), m.v ± s.d.
FC-1	0.12 ± 0.01	54.2 ± 0.11	58 ± 0.57	27 ± 1.76	1.43 ± 0.64	18 ± 0.25
FC-2	0.13 ± 0.03	58.8 ± 0.11	50 ± 0.57	28 ± 1.45	1.56 ± 0.43	14 ± 0.21
FC-3	0.14 ± 0.04	60.2 ± 0.17	37 ± 0.57	29 ± 2.55	1.67 ± 0.29	10 ± 0.32
FC-4	0.156± 0.00	69.9 ± 0.17	58 ± 1.15	33 ± 1.35	1.66 ± 0.52	19 ± 0.52
FC-5	0.14 ± 0.01	64.2 ± 0.05	28 ± 0.57	34 ± 1.16	1.82 ± 0.12	22 ± 0.16
FC-6	0.16 ± 0.04	72.1 ± 0.46	72 ± 0.57	31 ± 2.54	1.74 ± 0.24	19 ± 0.18
FC-7	0.17 ± 0.01	80.5 ± 0.11	102 ± 1.15	29 ± 6.93	1.65 ± 0.41	24 ± 0.28
FC-8	0.18 ± 0.05	82.2 ± 0.34	96 ± 0.57	31 ± 4.67	1.68 ± 0.03	21 ± 0.41
FC-9	0.19 ± 0.01	88.3 ± 0.05	90 ± 0.57	26 ± 3.88	1.87 ± 0.19	21 ± 0.51

**Table 4:** Pharmacokinetic parameters after oral administration of pure EBT and FC-5 orodispersible films (*n* = 6).

Formulations	C_max_ (ng/mL)	Tmax (h)	AUC_0–24_ (ng/mL/h)	t_1/2_ (h)
EBT	82.8	3	1242	4.2
FC-5	138.6	1	2713	3.6

**Table 5:** Change in body weight as a function of days following oral drug administration.

Groups	Body Weight (g), m.v. ± s.d.		
	1st Day	7th Day	14th Day
A	212.8 ± 6.2	216 ± 7.1	223 ± 6.6
B	214.2 ± 10.7	220.9 ± 8.4	227.4 ± 8.1

**Table 6:** Blood hematological analysis of control and tested rat groups.

Hematological Parameters	Group A	Group B
Hb g/dL	12	11.4
Hct (%)	42.3	43.8
WBCs × 10^6^ /L	5.8	5.7
RBCs × 10^6^/mm^3^	5.88	5.52
Platelets × 10^9^/L	272	257
Monocytes (%)	7	6
Neutrophils (%)	40	54
Lymphocytes (%)	50	43
MCV (%)	70.4	66.2
MCH pg/cell	20.4	19.6
MCHC (%)	30.8	28.1
Eosinophils × 10^3^ μL	3	3

**Table 7:** Blood biochemical analysis of control and tested groups of rats.

Biochemical Parameters	Group A	Group B
Bilirubin total mg/dL	0.8	0.7
Conjugated mg/dL	0.3	0.3
SGPT U/L	70	72
SGOT U/L	62	64
Alkaline Phosphatase U/L	104	102
Total Protein G/dL	9.6	9.7
Albumin G/dL	3	2.8
Globulins G/dL	6.8	7.1
A/G Ratio	0.5	0.4

## Data Availability

The data presented in this study are available on request from the corresponding author. The data are not publicly available as it was originally produced through research.

## Supplementary Materials

The following are available online at https://www.mdpi.com/1999-4923/13/1/54/s1, Figure S1. A few images of Poloxamer-188 and TPGS-1000 mixed micelles containing orodispersible sublingual films.
