# Supplementary material for: Poloxamer-188 and d-α-Tocopheryl Polyethylene Glycol Succinate (TPGS-1000) Mixed Micelles Integrated Orodispersible Sublingual Films to Improve Oral Bioavailability of Ebastine; In Vitro and In Vivo Characterization
Source: Pharmaceutics. 2021 Jan 4;13(1):54. doi: 10.3390/pharmaceutics13010054 (PMC7823785; doi:10.3390/pharmaceutics13010054)
Supplement: Supplementary file 1 [file pharmaceutics-13-00054-s001.pdf]

pharmaceutics

Supplementary Materials: Poloxamer-188 and D-α-Tocopheryl Polyethylene Glycol Succinate (TPGS-1000) Mixed Micelles Integrated Orodispersible Sublingual Films to Improve Oral Bioavailability of Ebastine; In Vitro and In Vivo Characterization

Nayyer Islam, Muhammad Irfan, Salah-Ud-Din Khan, Haroon Khalid Syed, Muhammad Shahid Iqbal, Ikram Ullah Khan, Amina Mahdy, Mohamed Raafat, Mohammad Akbar Hossain, Sana Inam, Rabia Munir and Memoona Ishtiaq

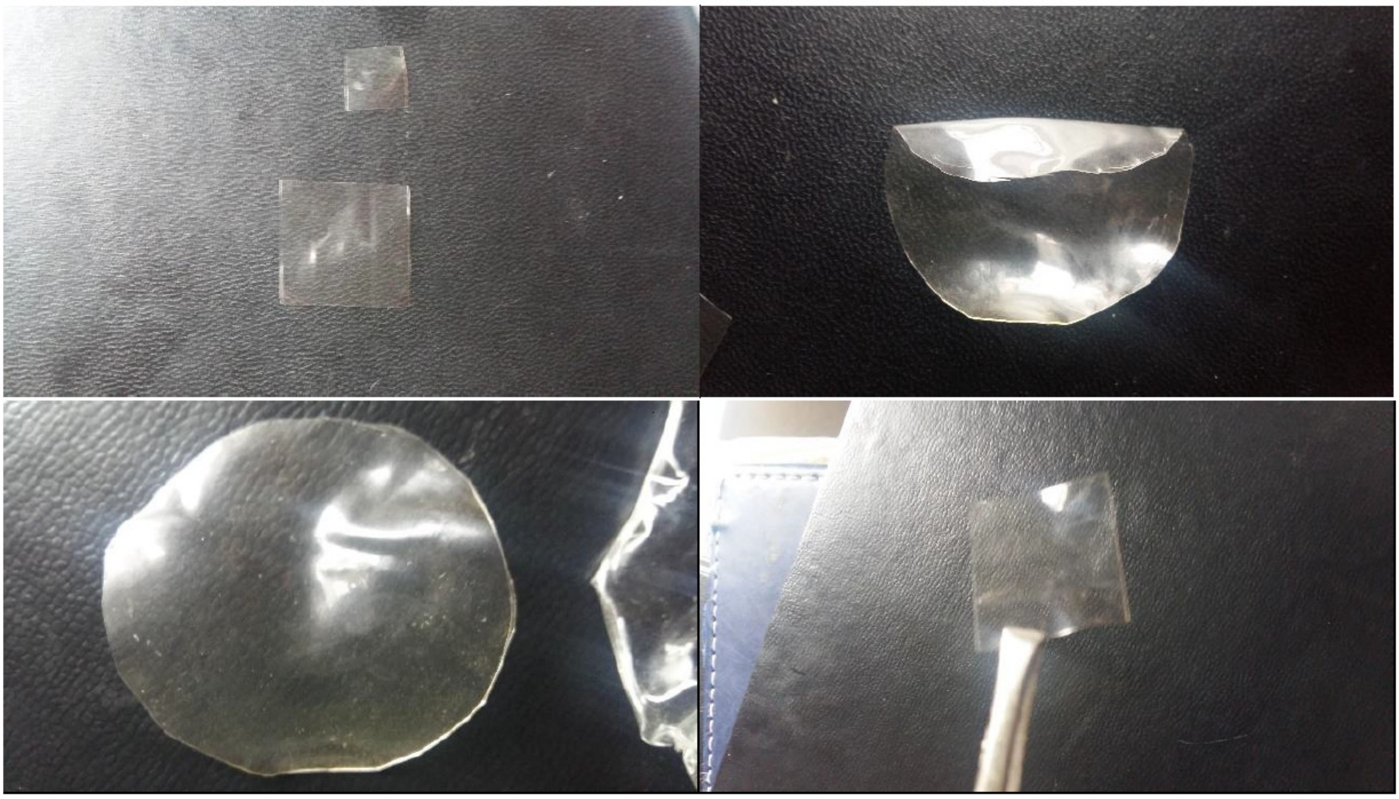

Figure S1. A few images of Poloxamer-188 and TPGS-1000 mixed micelles containing orodispersible sublingual films.
